# Interferon-free treatment for patients with chronic hepatitis C and autoimmune liver disease: higher SVR rates with special precautions for deterioration of autoimmune hepatitis

**DOI:** 10.18632/oncotarget.24391

**Published:** 2018-02-03

**Authors:** Tatsuo Kanda, Shin Yasui, Masato Nakamura, Shingo Nakamoto, Koji Takahashi, Shuang Wu, Reina Sasaki, Yuki Haga, Sadahisa Ogasawara, Tomoko Saito, Kazufumi Kobayashi, Soichiro Kiyono, Yoshihiko Ooka, Eiichiro Suzuki, Tetsuhiro Chiba, Hitoshi Maruyama, Fumio Imazeki, Mitsuhiko Moriyama, Naoya Kato

**Affiliations:** ^1^ Division of Gastroenterology and Hepatology, Department of Medicine, Nihon University School of Medicine, Tokyo 173-8610, Japan; ^2^ Department of Gastroenterology, Chiba University, Graduate School of Medicine, Chiba 260-8670, Japan

**Keywords:** hepatitis C virus, direct-acting antivirals, autoimmune hepatitis, primary biliary cholangitis, sustained virological response

## Abstract

**Background:**

Interferon-free treatment can achieve higher sustained virological response (SVR) rates, even in patients in whom hepatitis C virus (HCV) could not be eradicated in the interferon treatment era. Immune restoration in the liver is occasionally associated with HCV infection. We examined the safety and effects of interferon-free regimens on HCV patients with autoimmune liver diseases.

**Results:**

All 7 HCV patients with autoimmune hepatitis (AIH) completed treatment and achieved SVR. Three patients took prednisolone (PSL) at baseline, and 3 did not take PSL during interferon-free treatment. In one HCV patient with AIH and cirrhosis, PSL were not administered at baseline, but she needed to take 40 mg/day PSL at week 8 for liver dysfunction. She also complained back pain and was diagnosed with vasospastic angina by coronary angiography at week 11. However, she completed interferon-free treatment. All 5 HCV patients with primary biliary cholangitis (PBC) completed treatment and achieved SVR. Three of these HCV patients with PBC were treated with UDCA during interferon-free treatment.

**Conclusions:**

Interferon-free regimens could result in higher SVR rates in HCV patients with autoimmune liver diseases. As interferon-free treatment for HCV may have an effect on hepatic immunity and activity of the autoimmune liver diseases, careful attention should be paid to unexpected adverse events in their treatments.

**Methods:**

Total 12 patients with HCV and autoimmune liver diseases [7 AIH and PBC], who were treated with interferon-free regimens, were retrospectively analyzed.

## INTRODUCTION

Hepatitis C virus (HCV) causes chronic and persistent infection and is an important etiologic factor of advanced fibrosis/cirrhosis and hepatocellular carcinoma (HCC) [1]. Eradication of HCV could reduce the risk of the occurrence of HCC and liver-related deaths as well as liver-unrelated deaths, although it is difficult to eliminate HCV [2]. Interferon-free treatment can achieve higher sustained virological response (SVR) rates, even in patients in whom HCV could not be eradicated in the interferon treatment era [3].

Autoimmune hepatitis (AIH) and primary biliary cholangitis (PBC) are representative autoimmune liver diseases [4, 5]. AIH possesses a chronic active hepatitis histologically with autoimmune features and poor prognosis without immune suppressive therapies [4, 6]. PBC is diagnosed based on the presence of an antimitochondrial antibody (AMA), elevated cholestatic liver enzyme and/or histologically by chronic non-suppurative destructive cholangitis (CNSDC) [5, 7].

Patients with autoimmune liver diseases and hepatitis C virus (HCV) infection are occasionally observed [8, 9]. Patients with autoimmune hepatitis (AIH) and HCV infection often have advanced liver diseases at initial clinical and histological assessments [8]. PBC diagnosis in HCV patients is difficult and usually delayed [9]. Those with HCV and PBC do not have better outcomes than those with only HCV [9].

Immune restoration in the liver is occasionally associated with HCV infection [10, 11]. Studies using interferon-free regimens demonstrated an immune restoration of immunologic responses after HCV clearance [12–14]. Thus, HCV clearance has effects on the human immunologic response. HCV patients with autoimmune liver diseases could not be treated by interferon-including regimens because such regimens were contraindicated in these patients in the interferon era [15]. In the present study, we retrospectively examined the safety and effects of interferon-free regimens on HCV-patients with autoimmune liver diseases.

## RESULTS

### Treatment response in HCV patients with AIH

**Figure 1:**
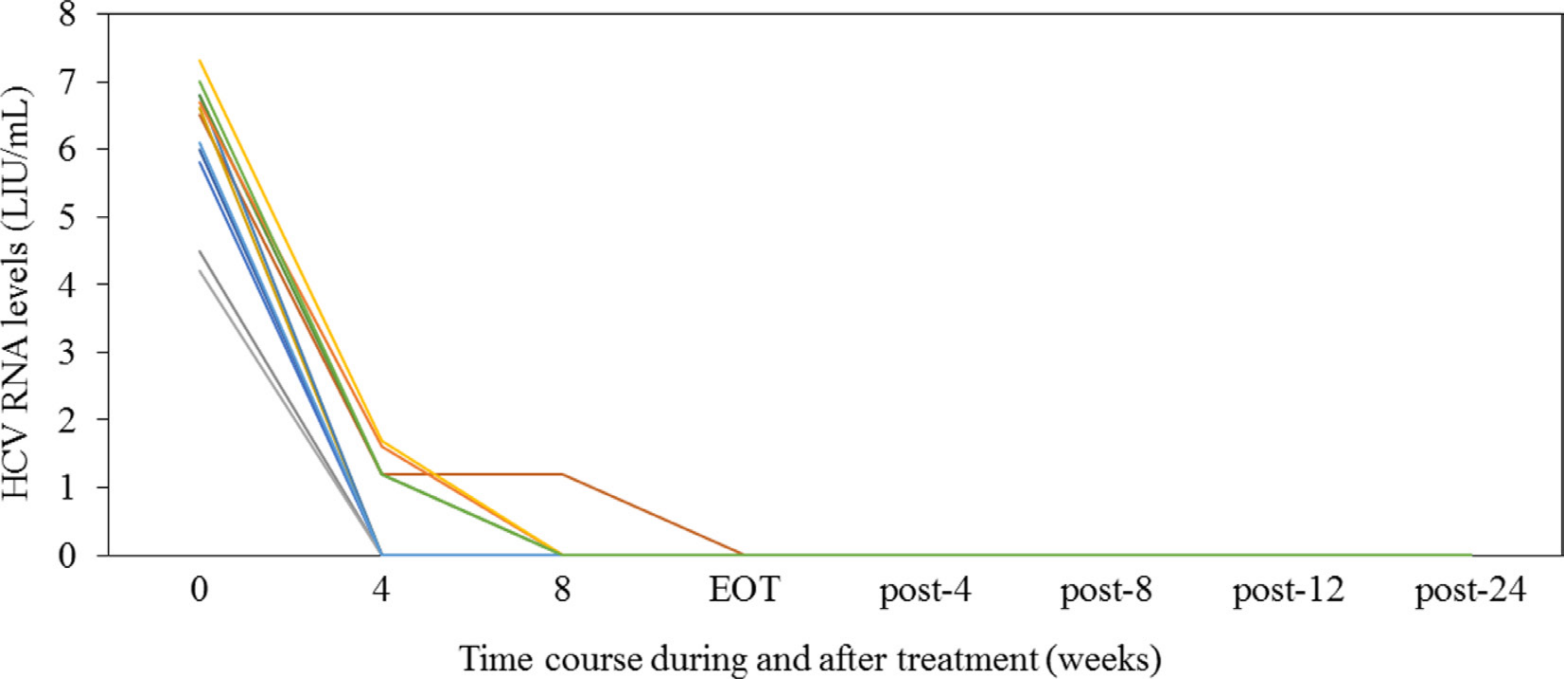
Serum HCV RNA levels (LIU/mL) during and after interferon-free treatment in the present study EOT, end of the treatment response.

**Figure 2:**
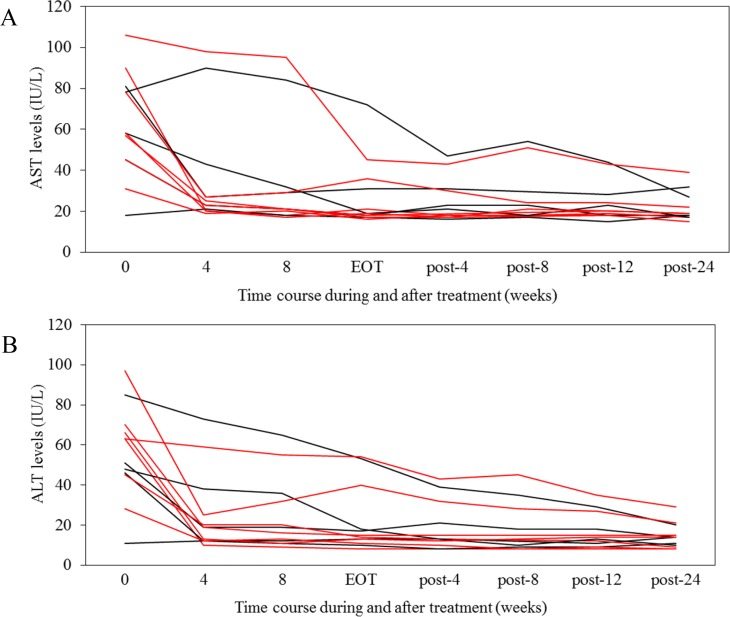
Serum transaminase levels during and after interferon free treatment (**A**) Aspartate transaminase (AST) (IU/L). (**B**) Alanine transaminase (ALT) (IU/L). AIH, red line; PBC, black line. EOT, end of the treatment response.

All 12 patients with autoimmune liver diseases completed treatment and achieved SVR (Figures 1 and 2). In the 7 patients with AIH, 4 and 3 were HCV genotypes (GTs) 1 and 2, respectively, and 3, 1 and 3 patients were treated with SOF/LDV, DCV/ASV and SOF/RBV, respectively. In 50% (2/4) of HCV GT-1 and 33% (1/3) of HCV GT-2 patients, RVR was achieved (Table 1 and Figure 1). Four patients with AIH and non-RVR achieved HCV RNA negativity at week 8. Three patients (nos. 6, 7 and 8) took prednisolone (PSL) at baseline, and 4 (nos. 9, 10, 11 and 12) did not take PSL at baseline.

Table 1Patient characteristics

**Table:** 

No.	PBC/AIH	Age (y) /Sex	HCV GTs	HCV RNA (LIU/mL)	PH of IFNs	BMI (kg /m^2^)	Liver biopsy findings	LS (kPa)	Concomitant Drugs: UDCA (mg/d) /PSL (mg/d) /Aza (mg/d)
1	PBC	58/F	1b	6	Naïve	20.7	N.D.	6.6	600/0/0
2	PBC	79/F	1b	6.5	Naïve	16.7	N.D.	10.5	0/0/0
3	PBC	80/M	1b	4.5	Naïve	19.4	N.D.	7.7	600/0/0
4	PBC	62/F	1b	6.6	Naïve	21.7	N.D.	6.2	0/0/0
5	PBC	57/F	1b	6.8	Naïve	27.1	Stage 2 CNSDC+ Vanishing bile duct (+)	10.4	900/0/0
6	AIH	77/F	1b	6.8	Naïve	24.5	F2/A3	N.D.	900/5/0
7	AIH	72/F	1b	5.8	Naïve	21.6	F3/A3	N.D.	600/10/0
8	AIH	69/F	1b	6.7	Naïve	22.9	F2/A2	9.3	600/5/25
9	AIH	59/F	1b	4.2	Naïve	22.8	F4/A3	N.D.	0/40(8W-)/0
10	AIH	71/F	2a	7.3	Naïve	21	N.D.	N.D.	600/0/0
11	AIH	62/M	2a	6.1	Naïve	21.3	F2/A3	15.1	600/0/0
12	AIH	73/M	2b	7	PR-Null	23.1	F3/A3	12.6	0/0/0

PBC, primary biliary cholangitis; AIH, autoimmune hepatitis; GTs, genotypes; PH of IFNs: past medical history of interferon treatment, BMI, body mass index; LS, liver stiffness; UDCA, ursodeoxycholic acid; PSL, prednisone; Aza, azathioprine; N.D., not done

**Table:** 

No.	ANA (−fold)	AMA M2 (U/mL)	ASMA (−fold)	LKM-1	IgG (mg/dL)	IgM (mg/dL)	AST (IU/L)	ALT (IU/L) />500	PLT (× 10^4^/μL)	Tx
1	Neg	156	Neg	Neg	2946	368	18	10/−	17.2	SOF/LDV
2	Neg	16.2	Neg	Neg	1518	115	74	44/−	9.2	SOF/LDV
3	80	10.8	Neg	Neg	1953	82	78	85/−	17	SOF/LDV
4	Neg	141	Neg	Neg	1486	280	45	51/−	19	SOF/LDV
5	Neg	218	Neg	Neg	1615	101	63	68/−	13.8	OMV/PTV/r
6	160	Neg	Neg	Neg	1565	117	77	62/−	9.3	DCV/ASV
7	80	Neg	Neg	Neg	2308	248	43	44/−	20.2	SOF/LDV
8	160	Neg	Neg	Neg	1947	36	32	31/−	9.4	SOF/LDV
9	>1280	Neg	Neg	Neg	4304	127	107	61/−	10.9	SOF/LDV
10	Neg	Neg	Neg	Neg	1636	229	88	96/+	8.3	SOF/RBV
11	640	Neg	Neg	Neg	2208	160	50	56/+	17.7	SOF/RBV
12	80	Neg	Neg	63	2465	86	84	115/−	14.3	SOF/RBV

ANA, anti-nuclear antibody; AMA, anti-mitochondrial antibody; ASMA, anti-smooth muscle antibody; LKM-1, anti-liver/kidney microsome type 1 antibody; Ig, immunoglobulin; AST, aspartate aminotransferase; ALT, alanine aminotransferase; PLT, platelets; Tx, DAA treatment for HCV; Neg, negative; +/−, with or without experience; SOF, sofosbuvir; LDV, ledipasvir; OBV/PTV/r, ombitasvir/paritaprevir/ritonavir; DCV/ASV, daclatasvir/asunaprevir; RBV, ribavirin.

**Figure 3:**
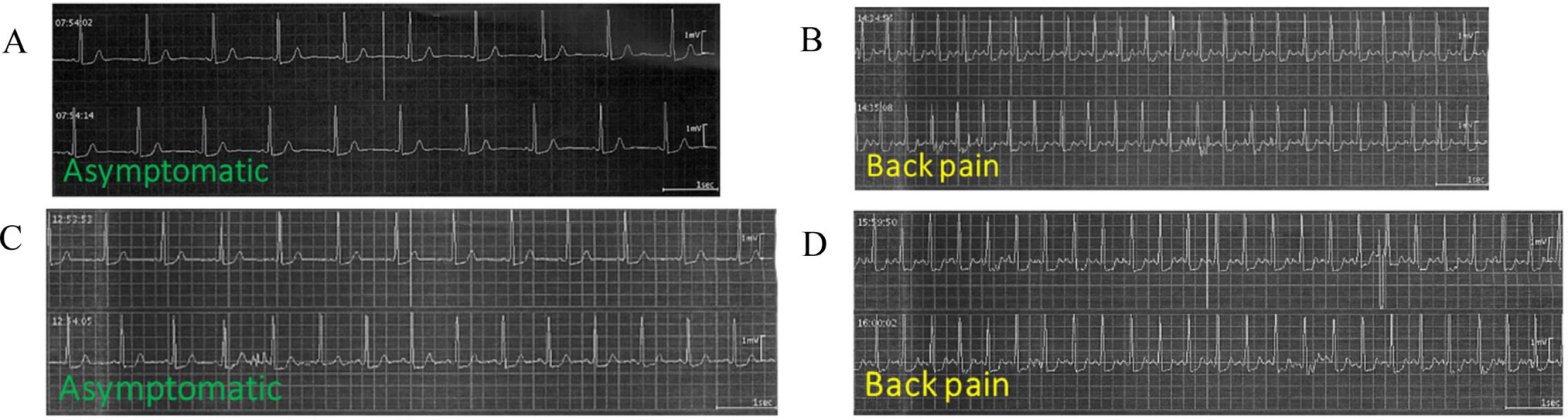
Electrocardiograms (ECGs) of patient no. 9 59-year female with chronic hepatitis (F4/A3), HCV and autoimmune liver diseases. She had hypertension. (**A**)Asymptomatic. (**B**) Backpain. (**C**) Asymptomatic. (**D**) Backpain.

In a female HCV GT-1-patient with AIH and cirrhosis (no. 9), PSL was not administered at baseline, but she needed to take 40 mg/day PSL at week 8 for liver dysfunction after HCV treatment. She complained of back pain and was diagnosed with vasospastic angina by coronary angiography at 11 weeks but completed her treatment (Figure 3).

Before treatment, in 7 HCV patients with AIH, 6 and 6 patients had abnormal aspartate aminotransferase (AST) and alanine aminotransferase (ALT) (more than 40 IU/L), respectively. At week 24 post-treatment, in all 7 of these patients, both AST and ALT were normalized.

### Treatment response in HCV patients with PBC

In 5 HCV GT-1 patients with PBC, 4 and 1 were treated with SOF/LDV and OBV/PTV/r, respectively, resulting in 75% (3/4) and 100% (1/1) RVR. One non-RVR patient achieved HCV RNA negativity at week 12. Three of 5 patients were treated with UDCA during HCV treatment (Table 1 and Figure 1).

Before treatment, in 5 HCV patients with PBC, 4 and 4 patients had abnormal AST and ALT (more than 40 IU/L), respectively. At week 24 post-treatment, in all 5 of these patients, both AST and ALT were normalized.

### Case presentation of a HCVGT-1 patient with AIH (no. 9)

Before commencement of SOF/LDV, she felt general fatigue, and her liver function tests showed ALT 61 IU/L, total bilirubin (T. Bil) 1.7 mg/dL, prothrombin time (PT) 47%, and immunoglobulin (Ig) G 4301 mg/dL. At week 8, co-administration of PSL was started for liver dysfunction (ALT 55 IU/L, T. Bil 2.2 mg/dL, PT 52%, and IgG 3743 mg dL). At week 11, back pain appeared, and electrocardiogram (ECG) showed ST-segment depression. She was diagnosed with vasospastic angina by coronary angiography but completed her treatment (Figure 3). Finally, SVR24 was achieved (ALT 29 IU/L, T. Bil 0.9 mg/dL, PT 104%, and IgG 1629 mg/dL). At present, she takes UDCA 600 mg/day and PSL 15 mg/day, and she is well.

## DISCUSSION

In 9 HCV GT-1-patients with autoimmune liver diseases, RVR rates, end of treatment response (EOT) rates and SVR rates were 66.7, 100 and 100%, respectively. In 3HCV GT-2-patients with autoimmune liver diseases, RVR rates, EOT rates and SVR rates were 33.3, 100 and 100%, respectively. The present study found higher SVR rates in HCV patients with autoimmune liver diseases.

Concerning safety, in one patient with AIH, PSL was needed for liver dysfunction during the treatment of SOF/LDV. If liver dysfunction is due to autoimmune liver diseases, it may be important to treat autoimmune liver diseases before or during interferon-free treatment for HCV. Unexpectedly, in this case, vasospastic angina also developed as a complication during the treatment of SOF/LDV. Recently, Nirei *et al.* [16] reported ventricular tachycardia as a complication of SOF/LDV treatment. We do not know the association between the use of SOF/LDV and vasospastic angina.

Vento *et al.* [10] reported two patients with HCV who developed fulminant liver failure after stopping chemotherapy, indicating an immune-mediated mechanism for hepatocyte damage in HCV infection. These patients with liver failure seem to have developed immune restoration in the liver after stopping chemotherapy. HCV-positive mothers with increased ALT in the post-partum period, also presented a significant decrease in serum HCV RNA in the post-delivery period, and this event was concomitant with an increase in Th1 cytokine levels [11].

In the present study, one patients with AIH (no. 12) was classified into the AIH type 2, which is positive for anti-liver/kidney microsome type 1 (anti-LKM1) antibody. It has been reported that anti-LKM-1 was occasionally positive in patients with chronic active hepatitis C [17]. Further studies about the effects of DAAs on this type AIH will be needed.

Restoration of immunologic responses after HCV clearance with interferon-free treatment has also been reported [12–14]. Hepatitis B reactivation during or after interferon-free treatment for HCV may be associated with the restoration of adaptive and innate immune responses [12–14, 18, 19]. Thus, interferon-free treatment for HCV may have an effect on hepatic immunity and activity of autoimmune liver diseases. Exacerbation of AIH and PBC during the interferon-including regimens for chronic HCV infection has been observed [20–22]. Although interferon-free regimens seem safer than interferon-including regimens in patients with autoimmune liver diseases, clinician should pay a special attention to acute exacerbation of liver diseases and unexpected adverse events.

## CONCLUSIONS

The interferon-free regimens were relatively safe and effective for HCV-infected patients with autoimmune liver diseases. Some patients with active AIH may require immune therapy before HCV treatment. Close monitoring and careful attention are needed to handle unexpected adverse events.

## PATIENTS AND METHODS

### Patients

A total of 467 HCV-infected patients, some of whom were described previously [23–25], started treatment with interferon-free treatment by 2016 at the Department of Gastroenterology, Chiba University Hospital. Among them, 12 patients with autoimmune liver diseases (7 AIH and 5 PBC) were analyzed [25% male, mean age 68±8.4 years, 92% treatment-naive, 75% GT-1b, 33% cirrhosis] (Table 1). This retrospective study was approved by Ethics Committee of Chiba University School of Medicine (numbers 1462 and 1753). Participants in the study were recruited from Chiba University Hospital. This study was conducted in accordance with the Declaration of Helsinki.

### Diagnosis of AIH and PBC

AIH is diagnosed by the international autoimmune hepatitis criteria for the diagnosis of autoimmune hepatitis [26, 27]. PBC was diagnosed based on the presence of AMA, elevated cholestatic liver enzyme and/or histologically by CNSDC as described previously [5, 7].

### Treatment protocol

In total, 9 HCV GT-1b patients were included: 7, 1 and 1 were treated with sofosbuvir/ledipasvir (SOF/LDV) for 12 weeks, ombitasvir/paritaprevir/ritonavir (OBV/PTV/r) for 12 weeks and daclatasvir/asunaprevir (DCV/ASV) for 24 weeks, respectively. All 3 HCV GT-2 patients were treated with SOF/ribavirin (RBV) for 12 weeks (Table 1). Before the use of OBV/PTV/r or DCV/ASV, HCV NS5A resistance associated variants (RAVs) were excluded as described previously [23].

### Laboratory tests

Laboratory tests were measured by standard laboratory techniques at a central laboratory in Chiba University Hospital. Blood samples were obtained at the baseline, at weeks 4, 8 and 12 of treatment, and then 4, 8, 12 and 24 weeks after the end of treatment [24].

### Measurement of HCV RNA

HCV RNA was measured by COBAS TaqMan HCV assay version 2.0 (Roche Diagnostics, Tokyo, Japan), with a lower limit of quantification of 15 IU/mL. We used the definition of rapid virological response (RVR), consisting of HCV RNA <15 IU/mL at week 4 according to the 2014 EASL guidelines. SVR was defined as negativity of HCV RNA at 24 weeks after stopping treatment. HCV genotyping was performed as previously described [24].

### Statistical analysis

Data are expressed as the mean ± standard deviation (SD). Statistical analyses were performed by univariate analyses using Student’s *t*-test or the chi-square test. *p* < 0.05 was considered statistically significant. Statistical analysis was performed using Excel Statistics program for Windows 2010 (SSRI, Tokyo, Japan) and DA Stats software (O. Nagata, Nifty Serve: PAF01644).
